# *PLOS Biology* at 20: Exploring possible futures

**DOI:** 10.1371/journal.pbio.3002377

**Published:** 2023-10-19

**Authors:** Nonia Pariente, on behalf of the PLOS Biology staff editors

**Affiliations:** Public Library of Science, San Francisco, California, United States of America and Cambridge, United Kingdom

## Abstract

Twenty years ago this month, PLOS Biology was launched, helping to catalyze a movement that has transformed publishing in the life sciences. This Editorial introduces our October issue, in which we explore how the community can continue innovating for positive change in the next decades.

## Teaser

Twenty years ago this month, *PLOS Biology* was launched, helping to catalyze a movement that has transformed publishing in the life sciences. In this issue, we explore how the community can continue innovating for positive change in the next decades.

This article is part of the *PLOS Biology* 20th Anniversary Collection.

Twenty years ago this month, *PLOS Biology* opened its digital doors to start a revolution in the way life science research is communicated to the world [1]. Throughout this year, we have been celebrating 20 years of publishing life science research. We’ve done so through Perspectives that either take stock of the past 2 decades of research and look at what might come next in fields across the life sciences, or ones that look at past *PLOS Biology* papers through the lens of time, reflecting on how they contributed to their fields. We have also been hearing about the impact that the journal has had on researchers; in a Biologue post, Lisa Maier shared how a *PLOS Biology* paper [2] set the course for her research career. We invite you to take a look at our 20th Anniversary Collection and enjoy the celebration.

This month, to mark 20 years since our first issue, we feature a series of articles on scientific publishing and open science, aiming to spark conversations around what the next decades will hold. The research ecosystem is in flux, with publishing (perhaps) catching up to the digital age, and with increasing calls for openness, rigor, and accountability. It is up to all of us—researchers, funders, hiring institutions, publishers—to work toward a fair and accessible system that incentivizes scientific advance over individual recognition. At the beginning of the year, we outlined some of the challenges ahead [3]; in this issue, we hope you find food for thought and inspiration to enact positive change.

*PLOS Biology* has been pioneering in more ways than one. At a time when leadership positions at scientific journals, throughout the scientific enterprise, and in society at large, have been dominated by men, *PLOS Biology* has been led by a diverse group of women since its launch. In this month’s opening editorial, Hemai Parthasarathy, Theodora Bloom, and Emma Ganley provide a glimpse into the launch and evolution of the journal, discussing our mission and vision over time [4].

In an Essay, Richard Sever takes us on a well-researched journey through the past, present, and possible futures of scientific publishing [5]. Grounded in the reality of the current publishing landscape and infrastructure, Richard outlines potential future scenarios for the dissemination of research findings that take full advantage of the digital age and are “…centered on making new findings available as soon as possible to spur further research and speed up its translation to actionable tools and knowledge that benefits society.”

A Perspective by Veronique Kiermer discusses current authorship practices and why they currently fail open science, outlining opportunities for improvement [6]. It is ironic that at a time of increasing calls for open science, contributions that enable open science are excluded from authorship in widely adopted guidelines. For example, between 2019 and 2022, a third of *PLOS Biology* authors did not report having drafted or edited the article, which is a requirement of the ICMJE authorship guidelines, but instead reported crucial contributions like data analysis, data curation, methodology, or software development. We have now adopted a different authorship policy that supports recognition of all meaningful contributions, while ensuring accountability for the research post-publication.

As Veronique says, “[i]t does not serve us well to reduce everything to articles as the only valuable output of research” [6]—we would do well to move to a system that values (and cites) other outputs such as software, data, and materials. This is a point that is also made in an Essay by Robert Thibault, Natascha Drude and colleagues, who write about the future of open science [7], including a whistle-stop tour of the current open science landscape and their vision for its future. They consider that open science needs to evolve towards an ecosystem in which transparency and openness permeate the research process from the start, enabling more rigorous and collaborative research. One of their recommendations to get there is doing more meta-research to identify and test solutions to current problems in the research ecosystem.

A similar call for meta-research is made in a Perspective by Tony Ross-Hellauer and colleagues, who argue that additional evidence is needed to inform implementation of open peer review practices. These are gaining traction without having been adequately assessed for efficacy, possible undesirable side effects, and the extent to which they affect the quality and trustworthiness of the items under review [8].

On the topic to moving open science practices more upstream in the research process, Manoj Lalu and colleagues propose that preregistration of animal studies be coupled with institutional animal ethics reviews that grant permission to perform the research [9]. This process, akin to the registration of clinical trials, would improve research transparency, reduce redundancy, and decrease selective reporting of research results.

Last but proverbially not least, a Perspective by Suzannah Rihn and Alexander Harms [10] discusses the important issue of how and why we should share biological materials generated through research. Both of them have recent positive experiences of sharing materials with the community [11,12] and note that sharing enables faster scientific advance, fosters collaboration, and contributes to addressing the reproducibility crisis, while preventing resource waste.

We hope this collection will inspire you to discuss in your networks where you as a community would like to go and motivate you to take the necessary action to effect change. We’d like to be your partners in that journey. Feel free to reach out with any feedback—we’d love to hear from you!
